# Supplementary material for: Iterative improvement in the automatic modular design of robot swarms
Source: PeerJ Comput Sci. 2020 Dec 7;6:e322. doi: 10.7717/peerj-cs.322 (PMC7924708; doi:10.7717/peerj-cs.322)
Supplement: Supplemental Information 3 [file peerj-cs-06-322-s003.zip › argos3/doc/api/standalone/a00437_source.html]

ARGoS: plugins/robots/foot-bot/control\_interface/ci\_footbot\_distance\_scanner\_actuator.h Source File


- Main Page
- Related Pages
- Namespaces
- Classes
- Files

- File List
- File Members

# plugins/robots/foot-bot/control\_interface/ci\_footbot\_distance\_scanner\_actuator.h

Go to the documentation of this file.

```
00001 
00042 #ifndef CCI_FOOTBOT_DISTANCE_SCANNER_ACTUATOR_H
00043 #define CCI_FOOTBOT_DISTANCE_SCANNER_ACTUATOR_H
00044 
00045 /* To avoid dependency problems when including */
00046 namespace argos {
00047    class CCI_FootBotDistanceScannerActuator;
00048 };
00049 
00050 #include <argos3/core/control_interface/ci_actuator.h>
00051 #include <argos3/core/utility/math/angles.h>
00052 
00053 namespace argos {
00054 
00055    class CCI_FootBotDistanceScannerActuator: virtual public CCI_Actuator {
00056 
00057    public:
00058 
00062       virtual ~CCI_FootBotDistanceScannerActuator() {}
00063 
00072       virtual void SetAngle(const CRadians& c_angle) = 0;
00073 
00083       virtual void SetRPM(Real f_rpm) = 0;
00084 
00088       virtual void Enable() = 0;
00089 
00093       virtual void Disable() = 0;
00094 
00095 #ifdef ARGOS_WITH_LUA
00096       virtual void CreateLuaState(lua_State* pt_lua_state);
00097 #endif
00098 
00099    };
00100 
00101 };
00102 
00103 #endif
```

---

Generated on 10 Jul 2018 for ARGoS by 
 1.6.1
